# Impact of Prior Use of Four Preventive Medications on Outcomes in Patients Hospitalized for Acute Coronary Syndrome--Results from CPACS-2 Study

**DOI:** 10.1371/journal.pone.0163068

**Published:** 2016-09-14

**Authors:** Min Li, Yubei Huang, Xin Du, Shenshen Li, Jiachao Ji, Anushka Patel, Runlin Gao, Yangfeng Wu

**Affiliations:** 1 Department of Epidemiology and Biostatistics, School of Public Health, Peking University Health Science Center, Beijing, China; 2 Department of Epidemiology and Biostatistics, Tianjin Medical University Cancer Institute and Hospital, Tianjin, China; 3 The George Institute for Global Health at Peking University Health Science Center, Beijing, China; 4 Beijing Anzhen Hospital, Capital Medical University, Beijing, China; 5 The George Institute for Global Health, University of Sydney, Sydney, Australia; 6 The Department of Cardiology, Cardiovascular Institute and Fuwai Hospital, Chinese Academy of Medical Sciences and Peking Union Medical College, Bejing, China; Osaka University Graduate School of Medicine, JAPAN

## Abstract

**Background:**

It is widely reported that long-term use of four preventive medications (antiplatelet agents, angiotensin converting enzyme inhibitor / angiotensin receptor blocker, statin and beta-blockers) reduce the risk of subsequent acute coronary syndromes (ACS). It is unclear whether these four medications benefit patients who develop ACS despite its use.

**Methods and Results:**

Logistic regression and propensity-score was applied among 14790 ACS patients to assess the association between prior use of four preventive medications and in-hospital outcomes including severity of disease at presentation (type of ACS, systolic blood pressure <90 mmHg, and heart rate> = 100 beats/min), complicating arrhythmia and major adverse cardiovascular events (MACEs, including all deaths, non-fatal myocardial infarction or re-infarction, and non-fatal stroke). Prior use of each of the four medications was significantly associated with less severity of disease (ORs ranged from 0.40 to 0.82, all P<0.05), less arrhythmia (ORs ranged from 0.45 to 0.64, all P<0.05), and reduced risk of MACEs (ORs ranged from 0.59 to 0.73, all P<0.05) during hospitalization. Multiple variable-adjusted ORs of MACEs were 0.77, 0.67, 0.48 and 0.59 respectively in patients with 1, 2, 3 and 4 medications in comparison with patients with none, and other clinical outcomes showed the same trend (P for trend < 0.05).

**Conclusions:**

Among ACS patients in our study, those with prior use of four preventive medications presented with less disease severity, developed less arrhythmia and had a lower risk of in-hospital MACEs. The value of taking these medications may beyond just preventing occurrence of the disease.

## Introduction

Acute coronary syndromes (ACS) are highly prevalent conditions, and a major contributor to global mortality. On the basis of substantial evidence from large scale randomized trials, guidelines worldwide advocate the use of four preventive medications [antiplatelet agents (aspirin or clopidogrel), angiotensin-converting enzyme inhibitor (ACEI) or angiotensin receptor blocker (ARB), statin and beta-blockers] for ACS patients either in or after the acute phase, unless there are contraindications.

However, while the role of these medications in prevention from the incidence of ACS and other cardiovascular disease is well-established, for those who still developed ACS while taking these medications it remains largely unknown if there are also benefits from use of these medications. Previous studies have had conflicting findings with respect to the association between prior use of the four medications and subsequent clinical outcomes in patients who develop ACS despite its use [1–10].

Further, those studies mostly used mortality as study outcomes. It remains largely unknown that if prior use of these four medications would also impact on disease severity at presentation and development of complications during hospitalization. In addition, none of the previous studies examined all four medications and all in-hospital major clinical outcomes. The knowledge gained in the relationship of prior use of prevention medications and in-hospital major clinical outcomes in patients who develop ACS despite its use would help better understand the clinical effects of these medications, and may further emphasize the importance of appropriate prescription of and adherence to preventive medications in relevant patients. This study evaluated the relationship of prior use of the four preventive medications with in-hospital major clinical outcomes in a large cohort of ACS patients in China.

## Materials and Methods

### Study population

The study population was drawn from the Clinical Pathways for Acute Coronary Syndromes—Phase 2 (CPACS-2) Study [11–12]. Briefly, CPACS-2 was both a planned prospective registry study and a cluster randomized trial that sought to provide rigorous evidence to inform the routine use of clinical pathways in the management of ACS in China. The study recruited 75 hospitals from 17 provinces and municipalities throughout China, including 27 level 2 hospitals (broadly defined as regional hospitals providing medical services to several communities) and 48 level 3 hospitals (broadly defined as hospitals providing high level specialist medical services to several geographic regions). Adult patients (aged> = 18 years) with a final diagnosis of ACS were included. A total of 15,141 consecutive ACS patients were recruited between December 2007 and October 2010. The medical charts of eligible patients were reviewed to collect requested information by centrally trained and certified research personnel who were not involved with the clinical care of the patients.

For the present analyses, patients with missing information on key variables were excluded, including 349 patients with missing one or more outcome variables information and 2 for missing age information. Therefore, a total of 14790 patients were included in the present analyses.

### Prior use of medications

The prior use of the four guideline-recommended medications in the study included antiplatelet agents (aspirin or clopidogrel), ACEI/ARB, statin and beta-blockers. Prior use of preventive medications was defined as using any of these four medications in the majority of 28 days before onset of ACS, including those long-term users. The information on the prior use of preventive medications was collected through an interview with patients instead of from reviewing the medical chart because the medical chart recording on this kind information in level 2 hospitals in China is often lacking.

### In-hospital clinical outcomes

The major in-hospital clinical outcomes in the study include: (1) ACS severity at presentation, including type of ACS (ST-segment elevation myocardial infarction (STEMI), vs. non ST-segment elevation acute coronary syndrome (NSTE-ACS), presented with hypotension (systolic blood pressure (SBP) <90 mmHg) or tachycardia (heart rate (HR)> = 100 beats/min). (2) Clinical complications: major arrhythmia. (3) Major adverse cardiovascular events (MACEs): including all-cause mortality, non-fatal new or reoccurred myocardial infarction (MI), and non-fatal stroke. We also included major bleeding as side effect in relation to prior use of antiplatelet medications. The detail definitions of outcomes are summarized in S1 Table.

### Statistical analyses

We used logistic regression model to estimate the effect of the prior medications on clinical outcomes. First, we built separate propensity score model for each individual drug, which is the conditional probability of taking prior medication, based on factors from clinical judgment and bivariate analysis, including demographic characteristics (age, sex), health insurance, history of cardiovascular disease (CVD, including MI, angina pectoris, heart failure, stroke / transient ischemic attack), risk factors of cardiovascular disease (current smoker, hypertension, diabetes), hospital level (level 2 or level 3) [13]. Then, we calculated the adjusted odd ratios (ORs) for each of the four medications, with the corresponding propensity score of the prior medication in the logistic model. To test the hypothesis that the effect of prior use of preventive medications may be mediated through the effect on disease severity at presentation, multiple logistic regression was performed, with both disease severity and the propensity score included as confounders.

Then, to better understand the effect of prior use of these medications alone or in combination, and at the same time coping with the significant co-linearity associations in prior use of these medications, we classified the study population according to the number of these prior medications and calculated ORs of the clinical outcomes for each group in comparison with those used none of these medications. The trend of risk with increase of number of the medications was tested using the same regression model and propensity score with the number of medications as a continuous variable. We also did the analysis separately for patients with and without history of CVD to understand if the purpose of prior use of these medications (as for primary or secondary prevention) modified the effects.

For the main analyses, we did not adjust for in-hospital treatment including reperfusion (percutaneous coronary intervention, coronary artery bypass grafting, thrombolytic) and medications (antiplatelet agents, ACEI/ARB, statin and beta-blockers) to avoid over adjustment, since it is unclear whether in-hospital treatments is administered to patients before the non-fatal outcomes happened, or used to cope with it. However, to test the stability of our finding, analyses with additional adjusted for in hospital treatments were performed as sensitivity analyses.

For all analyses, values of P <0.05 were considered to be statistically significant. All analyses were done using SAS version 9.3 (SAS Inc., Cary, NC, USA).

### Ethical standards

Ethics approval for CPACS-2 was obtained from the Human Research Ethics Committees at Cardiovascular Institute and Fuwai Hospital, Chinese Academy of Medical Sciences and Peking Union Medical College, Beijing, China and from the University of Sydney, Australia (Human Research Ethics Committees number: 09-2007/10276). All patients provided written informed consent prior to their inclusion in the study.

## Results

### General characteristics of study population and status in prior use of preventive medications

Table 1 detailed the baseline characteristics of all study participants by prior use of four recommended medications. The study patients were predominantly male and the mean age was 64 years. Patients on prior medications were slightly older, better insured, less likely to be a current smoker, and to receive thrombolytic therapy but significantly more likely having hypertension, diabetes, and history of MI, angina pectoris, stroke/ transient ischemic attack, heart failure, and to receive beta-blockers therapy. Among the four preventive medications, use of antiplatelet agents was most commonly reported (31.1%), while statins were least used (13.3%). A majority of the patients (61.7%) had no prior use of any of the four preventive medications. Even among those with history of cardiovascular disease, the proportion of no prior use of any of the four preventive medications was high (46.9%). (Characteristics of included and excluded patients are provided in S2 Table.)

**Table 1:** Baseline characteristics of ACS patients by prior use of four recommended medications.

Variables	Antiplatelet		ACEI/ARB		Statin		Beta-blockers	
	Yes	No	Yes	No	Yes	No	Yes	No
	(N = 4601)	(N = 10189)	(N = 2207)	(N = 12583)	(N = 1973)	(N = 12817)	(N = 2463)	(N = 12327)
Age, Mean (sd)	64.9(11.1)	63.4(12.2)*	65.4(11.0)	63.6(12.0)*	64.4(11.2)	63.8(11.9)*	64.2(11.2)	63.8(12.0)
Male	68.8	69.8	67.2	69.9*	69.5	69.5	67.2	70.0*
Health insurance	84.7	81.6*	86.0	82.0*	85.3	82.2*	84.4	82.2*
Risk factors of CVD								
Current smoker	24.0	34.3*	23.2	32.5*	22.4	32.4*	22.6	32.8*
Hypertension	65.2	53.8*	84.2	52.7*	64.5	56.3*	70.9	54.7*
Diabetes	25.3	18.3*	28.7	19.0*	26.7	19.5*	25.3	19.5*
History of CVD								
Myocardial infarction	25.5	6.3*	24.0	10.2*	31.2	9.3*	27.7	9.1*
Angina pectoris	62.3	31.8*	59.9	38.0*	65.6	37.6*	65.4	36.5*
Stroke/ transient ischemic attack	13.0	8.7*	13.5	9.5*	11.9	9.8*	11.1	9.9
Heart failure	10.0	4.5*	11.0	5.4*	10.8	5.5*	9.8	5.5*
Reperfusion therapy								
Percutaneous coronary intervention	46.2	46.8	45.6	46.8	49.5	46.2*	47.3	46.5
Coronary artery bypass grafting	1.3	0.6*	1.0	0.8	1.1	0.8	1.6	0.7*
Thrombolytic	2.7	7.9*	2.8	6.9*	2.6	6.8*	2.4	7.1*
In-hospital medications								
Antiplatelet	99.5	98.8*	99.0	99.0	99.2	99.0	99.1	99.0
ACEI/ARB	79.3	79.0	94.2	76.4*	79.5	79.0	80.4	78.8
Statin	93.8	93.8	94.3	93.7	97.9	93.2*	94.1	93.7
Beta-blockers	80.1	77.4*	82.6	77.5*	83.4	77.5*	93.1	75.3*

All data in the table are shown as %, except for age.

*P<0.05.

ACEI: angiotensin converting enzyme inhibitor; ACS: acute coronary syndrome; ARB: angiotensin receptor blocker; CVD: cardiovascular disease

### Prior use of four preventive medications with in-hospital clinical outcomes

#### 1) Association with severity of disease at presentation

Patients with prior use of any of the four medications showed a significant lower risk of presenting with STEMI rather than NSTE-ACS. They had significantly reduced risk of presenting with hypotension and tachycardia, even after adjustment for potential confounders (Table 2).

**Table 2:** Multiple clinical outcomes (%) by prior medications use and multi-variable adjusted ORs (95%CI) among ACS patients.

Outcome variables	Antiplatelet			ACEI/ARB			Statin			Beta-blockers		
	Yes	No	Adjusted OR ^a^ (95%CI)	Yes	No	Adjusted OR ^a^ (95%CI)	Yes	No	Adjusted OR ^a^ (95%CI)	Yes	No	Adjusted OR ^a^ (95%CI)
Severity at presentation												
STEMI subtype ^b^	23.8	46.6	0.50(0.46–0.54)	23.3	42.4	0.58(0.52–0.65)	20.3	42.5	0.47(0.42–0.53)	19.2	43.6	0.43(0.39–0.48)
SBP<90mmHg	1.0	2.2	0.53(0.38–0.75)	0.8	2.0	0.48(0.29–0.80)	0.8	2.0	0.49(0.29–0.83)	0.7	2.0	0.40(0.24–0.67)
HR> = 100bpm	6.5	9.4	0.69(0.60–0.80)	7.2	8.8	0.82(0.68–0.98)	5.0	9.1	0.56(0.45–0.69)	5.7	9.1	0.67(0.55–0.80)
Complications												
Arrhythmia	4.4	6.5	0.63(0.53–0.75)	4.0	6.2	0.64(0.50–0.80)	3.4	6.2	0.53(0.40–0.68)	3.0	6.4	0.45(0.35–0.58)
MACE												
Total MACEs	3.9	5.4	0.70(0.58–0.84)	3.4	5.2	0.59(0.46–0.76)	3.7	5.2	0.73(0.57–0.94)	3.5	5.3	0.70(0.55–0.89)
Death	2.1	3.4	0.62(0.49–0.79)	1.8	3.2	0.55(0.39–0.77)	1.6	3.2	0.52(0.36–0.76)	1.8	3.3	0.63(0.46–0.87)
Cardiac death	1.8	3.2	0.57(0.44–0.73)	1.5	3.0	0.48(0.33–0.70)	1.3	3.0	0.43(0.29–0.66)	1.6	3.0	0.58(0.41–0.82)
Non-fatal MI	1.1	1.6	0.64(0.45–0.90)	0.7	1.6	0.42(0.25–0.71)	1.3	1.4	0.91(0.59–1.40)	1.0	1.5	0.67(0.43–1.04)

^a^ Adjusted OR: adjusting for age, sex, insurance, history of CVD (myocardial infarction, angina pectoris, heart failure, stroke/ transient ischemic attack), risk factors of CVD (current smoker, hypertension, diabetes), and level of hospital.

^b^ STEMI compared to NSTE-ACS.

ACEI: angiotensin converting enzyme inhibitor; ACS: acute coronary syndrome; ARB: angiotensin receptor blocker; CI: confidence interval; CVD: cardiovascular disease; HR: heart rate; MACEs: major adverse cardiovascular events; MI: myocardial infarction; OR: odd ratio; SBP: systolic blood pressure; STEMI: ST-segment elevation myocardial infarction; NSTE-ACS: non ST-segment elevation acute coronary syndrome;

#### 2) Association with complications

Prior use of any of the four medications were also significantly associated with a lower risk of developing arrhythmia during hospitalization, and the association with arrhythmia was still significant for all four medications after adjusting for potential confounders.

#### 3) Association with MACEs

Prior use of any of the four medications was significantly associated with a reduced risk of any in-hospital MACEs, death, cardiac death. The associations were still significant after adjusting for potential confounders except for the associations between non-fatal MI and prior use of statin, and beta-blockers.

#### 4) Associations with MACEs and arrhythmia after further adjustment of disease severity at presentation

After adjusting for severity of disease at presentation, many of the associations became non-significant. The notable exception was for prior ACEI/ARB use, where associations remained significant (Table 3).

**Table 3:** ORs (95%CI) of prior medications use on in-hospital development of complications and MACEs among ACS patients after further adjusting for severity at presentation.

Outcome variables	Antiplatelet	ACEI/ARB	Statin	Beta-blockers
Complications				
Arrhythmia	0.84(0.71–1.01)	0.80(0.63–1.01)	0.73(0.56–0.96)	0.65(0.50–0.84)
Total MACEs				
MACEs	0.92(0.76–1.11)	0.72(0.56–0.94)	1.05(0.80–1.36)	1.01(0.79–1.30)
Death	0.82(0.64–1.05)	0.68(0.48–0.96)	0.77(0.52–1.13)	0.95(0.68–1.33)
Cardiac death	0.75(0.58–0.98)	0.60(0.41–0.88)	0.63(0.41–0.97)	0.89(0.63–1.27)
Non-fatal MI	0.88(0.62–1.25)	0.54(0.32–0.91)	1.36(0.87–2.11)	1.01(0.65–1.58)

All ORs were adjusting for age, sex, insurance, history of CVD (myocardial infarction, angina pectoris, heart failure, stroke / transient ischemic attack), risk factors of CVD (current smoker, hypertension, diabetes), hospital level plus severity at presentation (ACS subtype, SBP <90mmHg, HR> = 100bpm).

ACEI: angiotensin converting enzyme inhibitor; ACS: acute coronary syndrome; ARB: angiotensin receptor blocker; CI: confidence interval; CVD: cardiovascular disease; HR: heart rate; MACEs: major adverse cardiovascular events; MI: myocardial infarction; OR: odd ratio; SBP: systolic blood pressure;

#### 5) Association with severe bleeding and stroke

The ORs of developing hemorrhagic stroke were positively associated with prior use of antiplatelet agents and the association was still statistically significant after additional adjusting severity at presentation (Table 4).

**Table 4:** Bleeding (%) by prior antiplatelet agents use and multi-variable adjusted ORs (95%CI) among ACS patients (%).

Bleeding events	Prior use of antiplatelet, %		Adjusted OR ^a^ (95%CI)	Adjusted OR ^b^ (95%CI)
	Yes (N = 4601)	No (N = 10189)		
Major bleeding	2.0	1.5	1.33(1.00–1.77)	1.55(1.16–2.07)
Hemorrhagic stroke	0.6	0.2	2.96(1.56–5.62)	2.97(1.54–5.70)
Other bleedings	1.4	1.3	1.09(0.79–1.50)	1.31(0.94–1.82)

^a^ adjusting for age, sex, insurance, history of CVD (myocardial infarction, angina pectoris, heart failure, stroke/ transient ischemic attack), risk factors of CVD (current smoker, hypertension, diabetes), and level of hospital.

^b^ adjusting for age, sex, insurance, history of CVD (myocardial infarction, angina pectoris, heart failure, stroke / transient ischemic attack), risk factors of CVD (current smoker, hypertension, diabetes), level of hospital plus severity at presentation (ACS subtype, SBP <90mmHg, HR> = 100bpm).

ACS: acute coronary syndrome; CI: confidence interval; CVD: cardiovascular disease; HR: heart rate; OR: odd ratio; SBP: systolic blood pressure;

### Clinical outcomes in association with number of medications used in combination

**Fig 1:**
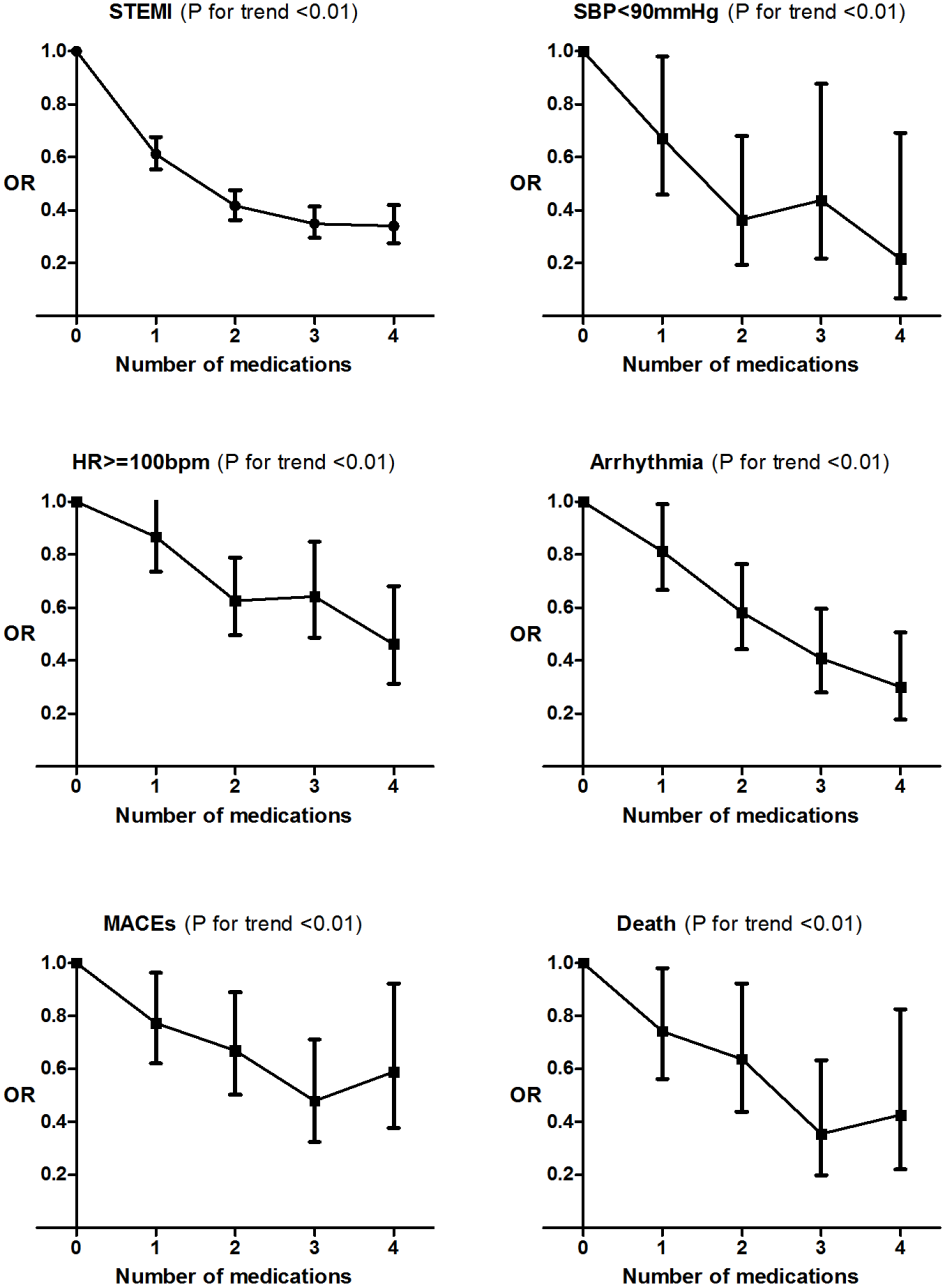
Trends in risk of multiple in-hospital clinical outcomes with increasing number of prior medications used. HR: heart rate; MACEs: major adverse cardiovascular events; OR: odd ratio; SBP: systolic blood pressure; STEMI: ST-segment elevation myocardial infarction;

Our data showed significant co-linearity of the four prior medications, with Pearson coefficient ranged from 0.3 to 0.5 (P<0.01). Thus, we analyzed the risk of clinical outcomes with the number of medications used. The risk of being presented with severity and developing arrhythmia and MACEs during hospitalization decreased significantly as the number of prior medication use was incrementally greater, from none to 4, even after adjusting for potential confounders (Fig 1).

### Results among patients with and without history of CVD

**Fig 2:**
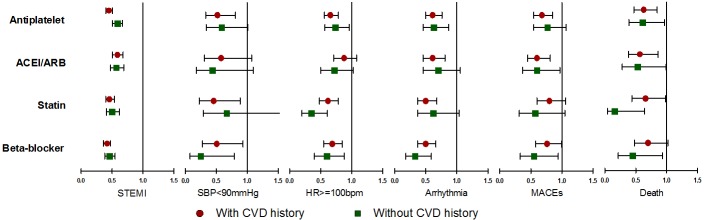
ORs (95%CI) of prior medications on in-hospital clinical outcomes among ACS patients, stratified by history of CVD. ACEI: angiotensin converting enzyme inhibitor; ACS: acute coronary syndrome; ARB: angiotensin receptor blocker; CI: confidence interval; CVD: cardiovascular disease; HR: heart rate; MACEs: major adverse cardiovascular events; OR: odd ratio; SBP: systolic blood pressure; STEMI: ST-segment elevation myocardial infarction;

We observed the above associations among patients with and without the history of CVD separately, and found that there were no significant differences except that 95% CI were generally wider (Fig 2), and the lines of trend with number of medications were fluctuant for patients without the history of CVD (S1 Fig).

### Sensitivity analyses

After additional adjusted for in-hospital treatments, ORs were calculated. The change was very minor and had no clear evidence to reverse our conclusions (S2–S5 Figs).

## Discussion

This large observational study of 14790 ACS patients showed that their prior use of antiplatelet agents, ACEI/ARB, statin and beta-blockers were significantly associated with lower severity of presenting ACS, a lower risk of developing in-hospital complications, including serious arrhythmia, major cardiovascular events and death. Beneficial impact of four prior medications on severity and complication was observed in patients with CVD history and patients without CVD history. Prior antiplatelet use was associated with greater risk of hemorrhagic stroke. With higher numbers of prior medication use, the risk of presenting with severity and developing arrhythmia and MACEs became significantly lower. And we found no differences in these associations between patients with and without history of CVD. These findings suggest that the benefits of these medications may extend beyond the known effect in prevention of occurrence of ACS, but may reduce the severity of disease and reduce in-hospital adverse outcomes in those who do develop an ACS anyway despite their prior use.

Our findings on the effects of antiplatelet agents were in concordance with GRACE (2002) and Medicare. In GRACE study, prior use of aspirin was not only associated with less likely to present with STEMI, but also reduced the incidence of in-hospital death significantly. In Medicare study, prior aspirin use lowered in-hospital and 6 month mortality [1–2]. In contrast, the TIMI, ESSENCE, PRISM-PLUS and PURSUIT studies suggested prior aspirin use did not yield benefit, and might slightly increase the incidence of death, recurrent ischemic events or emergency reperfusion therapy [3–6]. Several reasons could help to explain different findings. First, these studies were all clinical trials and the study participants were highly selected and very different from the patients in real world practice, such as study participants in our study and those from GRACE and Medicare. Second, the study aims of TIMI, ESSENCE, PRISM-PLUS and PURSUIT were to estimate effect of anticoagulants, though prior use of antiplatelet agents were neither listed in the exclusion criteria nor were wash-out periods required. Use of anticoagulant or fibrinolytic medications in the trials would greatly increase the risk of bleeding among patients with prior antiplatelet agent use, likely leading to significantly higher risks of hemorrhagic stroke or death. In these studies, the in-hospital rate of hemorrhagic stroke and other major bleeding were high, for example with 8.3% of patients without prior antiplatelet use experiencing moderate or severe bleeding in the PURSUIT trial. In fact, prior use of antiplatelet agents was significantly associated with in-hospital hemorrhagic stroke with a nearly three-fold higher risk in our study. But, because hemorrhagic stroke was a composition of MACEs, it could not change the conclusion on MACEs. In addition, the proportion of prior aspirin use was very high in TIMI-IIB, ESSENCE, PRISM-PLUS and PURSUIT, ranging from 59% in PRISM-PLUS study to 84% in TIMI-IIB study.

Few studies have reported the association of the prior use of other three medications with risk of developing clinical outcomes among ACS patients. GRACE (2004 and 2009) study found that prior statin use was associated with less diagnosis of acute MI among ACS patients, and lower incidence of in-hospital complications (congestive heart failure and severe arrhythmia), death, and MI recurrence [7–8]. Herlitz reported no significant correlation between prior beta-blockers and acute MI recurrence, death within 28 days among patients with suspected acute MI [9]. Singh demonstrated that the prior ACEI had no significant association with in-hospital death and MI [10]. Our study generally confirmed the findings from some previous studies but added new knowledge on the association with severity of disease at presentation and development of arrhythmia in hospital, and we adjusted more fully for possible confounders.

Unlike previous studies, we included all four preventive medications and observed the association of the risk of clinical outcomes with the number of medications used, rather than putting them into one model to adjust for their confounding effect for each other. Because they are often used together, and our data show significant co-linearity. Our finding that the number of medications showed a ‘dose-response’ relationship with the risk of adverse clinical outcomes suggests that all these four medications may have an effect in reducing the severity of the presenting ACS, and well as the risk of subsequent complications and early mortality.

To understand whether the effects observed on in-hospital events might be mediated by the severity of the presenting ACS, we further adjusted for the disease severity in the models to fit the association with these clinical endpoints. The results showed that all ORs approached closer to 1.0 and the associations of prior use of antiplatelet agents, statin and beta-blockers with MACEs became no longer significant. These findings suggest the beneficial effects of prior use of these medications may be mainly mediated through increasing the likelihood of developing a less severe ACS when this does occur. However, other mechanisms might be important in terms of prior statin, beta-blockers use and risk of arrhythmia, and the effects of prior ACEI/ARB more broadly. Although some studies also reveal patients on prior medication were more likely to present with UA as opposed to AMI, the mechanism is still unclear [14–15].

The interpretation of our results required caution due to the following limitations. First, this is an observational study and we cannot account for unmeasured and unknown confounders. Second, we excluded 351 patients from the study because of missing data on key variables. However, with multiple imputations, we found almost the same results (data not shown). Third, similar weight was given to all medications when looking at the combined effects. However, the benefits of each medications are all positive with similar OR values. Fourth, only ACS patients included may raises concerns of selection bias. However, the focus of our study is to reveal the effect of prior medications in patients developing an ACS anyway despite their prior use, not the effect to avert an ACS. As such, we believe that our work provides the best available assessment of the effects of prior medications use on the in-hospital course of ACS.

## Conclusions

In summary, among ACS patients in our study, those with prior use of four preventive medications presented with less disease severity, developed less arrhythmia and had a lower risk of in-hospital MACEs. The beneficial effect of prior use of these medications may be mainly mediated through increasing the likelihood of developing ACS with less severity even if it does occur. The findings indicated that these medications may have benefits beyond the prevention of occurrence of disease.

## Supporting Information

S1 Fig Trends in risk of multiple in-hospital clinical outcomes with increasing number of prior medications used, stratified by history of CVD. CVD: cardiovascular disease; HR: heart rate; MACEs: major adverse cardiovascular events; OR: odd ratio; SBP: systolic blood pressure; STEMI: ST-segment elevation myocardial infarction.
(TIF)

S2 Fig Multi-variable adjusted ORs of prior antiplatelet on arrhythmia and MACEs. The top part shows ORs in Table 2; the bottom part shows ORs with additional adjusted for in-hospital treatment including reperfusion therapy (thrombolytic therapy, percutaneous coronary intervention, coronary artery bypass grafting) and medications (antiplatelet agents, ACEI/ARB, beta-blockers, statin). ACEI: angiotensin converting enzyme inhibitor; ARB: angiotensin receptor blocker; MACEs: major adverse cardiovascular events; MI: myocardial infarction; OR: odd ratio.
(TIF)

S3 Fig Multi-variable adjusted ORs of prior ACEI/ARB on arrhythmia and MACEs. The top part shows ORs in Table 2; the bottom part shows ORs with additional adjusted for in-hospital treatment including reperfusion therapy (thrombolytic therapy, percutaneous coronary intervention, coronary artery bypass grafting) and medications (antiplatelet agents, ACEI/ARB, beta-blockers, statin). ACEI: angiotensin converting enzyme inhibitor; ARB: angiotensin receptor blocker; MACEs: major adverse cardiovascular events; MI: myocardial infarction; OR: odd ratio.
(TIF)

S4 Fig Multi-variable adjusted ORs of prior statin on arrhythmia and MACEs. The top part shows ORs in Table 2; the bottom part shows ORs with additional adjusted for in-hospital treatment including reperfusion therapy (thrombolytic therapy, percutaneous coronary intervention, coronary artery bypass grafting) and medications (antiplatelet agents, ACEI/ARB, beta-blockers, statin). ACEI: angiotensin converting enzyme inhibitor; ARB: angiotensin receptor blocker; MACEs: major adverse cardiovascular events; MI: myocardial infarction; OR: odd ratio.
(TIF)

S5 Fig Multi-variable adjusted ORs of prior beta-blockers on arrhythmia and MACEs. The top part shows ORs in Table 2; the bottom part shows ORs with additional adjusted for in-hospital treatment including reperfusion therapy (thrombolytic therapy, percutaneous coronary intervention, coronary artery bypass grafting) and medications (antiplatelet agents, ACEI/ARB, beta-blockers, statin). ACEI: angiotensin converting enzyme inhibitor; ARB: angiotensin receptor blocker; MACEs: major adverse cardiovascular events; MI: myocardial infarction; OR: odd ratio.
(TIF)

S1 Table Detail definitions of outcomes. (DOCX)

S2 Table Baseline characteristics of included and excluded ACS patients. (DOCX)
