# Supplementary figures and images for: Impact of Prior Use of Four Preventive Medications on Outcomes in Patients Hospitalized for Acute Coronary Syndrome--Results from CPACS-2 Study
Source: PLoS One. 2016 Sep 14;11(9):e0163068. doi: 10.1371/journal.pone.0163068 (PMC5023149; doi:10.1371/journal.pone.0163068)

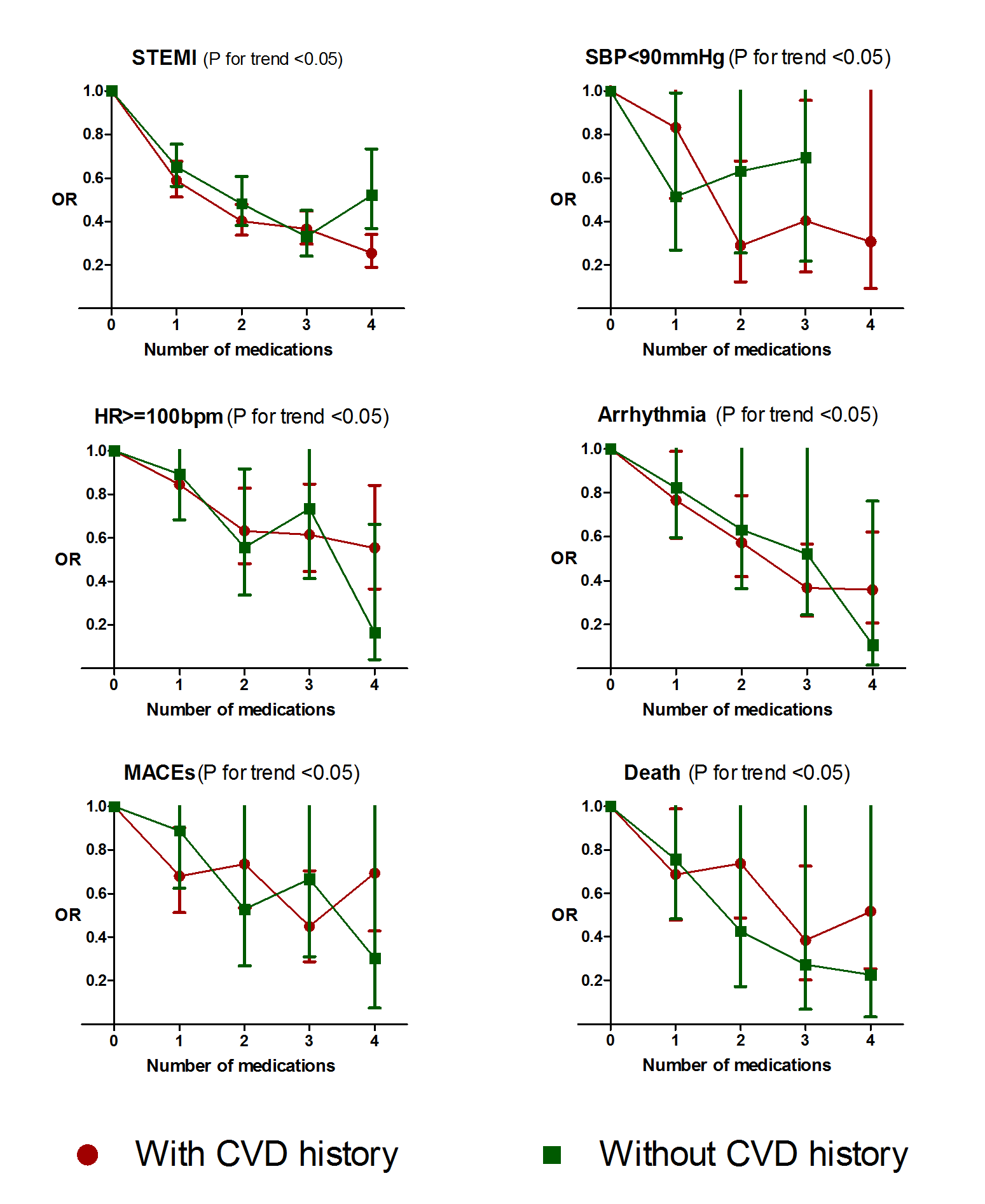

Supplement: S1 Fig — CVD: cardiovascular disease; HR: heart rate; MACEs: major adverse cardiovascular events; OR: odd ratio; SBP: systolic blood pressure; STEMI: ST-segment elevation myocardial infarction. (TIF) [file pone.0163068.s001.tif]

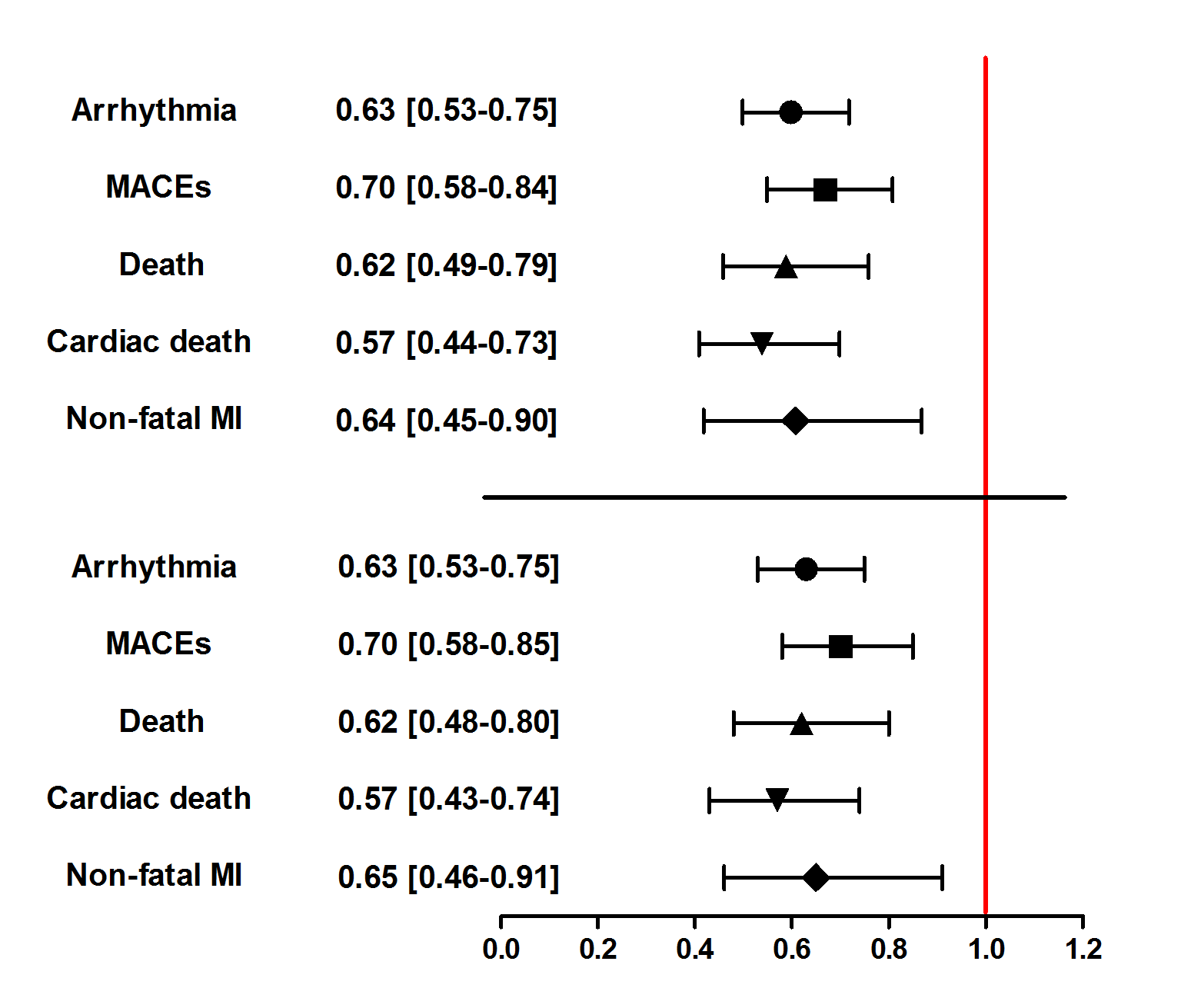

Supplement: S2 Fig — The top part shows ORs in Table 2; the bottom part shows ORs with additional adjusted for in-hospital treatment including reperfusion therapy (thrombolytic therapy, percutaneous coronary intervention, coronary artery bypass grafting) and medications (antiplatelet agents, ACEI/ARB, beta-blockers, statin). ACEI: angiotensin converting enzyme inhibitor; ARB: angiotensin receptor blocker; MACEs: major adverse cardiovascular events; MI: myocardial infarction; OR: odd ratio. (TIF) [file pone.0163068.s002.tif]

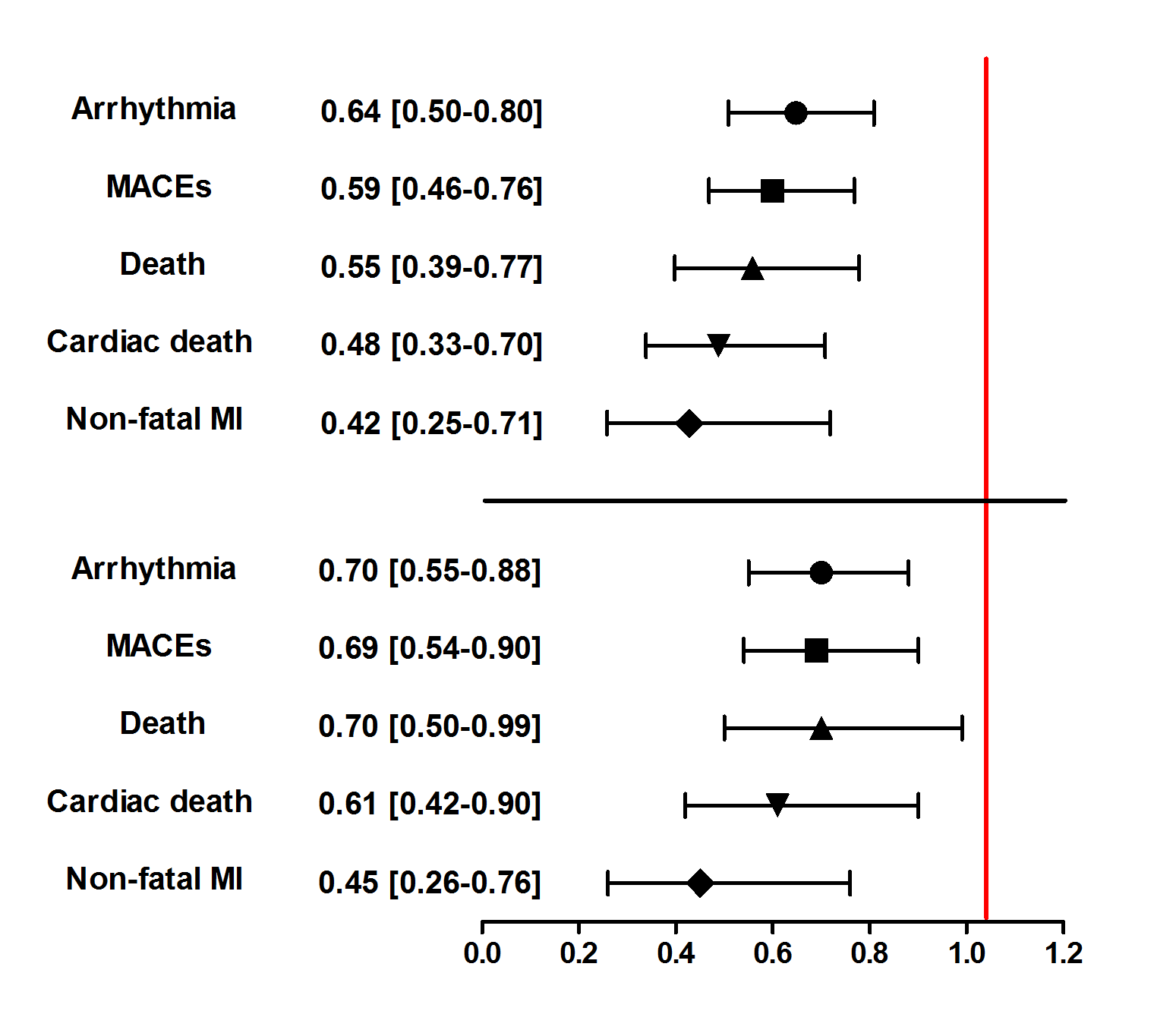

Supplement: S3 Fig — The top part shows ORs in Table 2; the bottom part shows ORs with additional adjusted for in-hospital treatment including reperfusion therapy (thrombolytic therapy, percutaneous coronary intervention, coronary artery bypass grafting) and medications (antiplatelet agents, ACEI/ARB, beta-blockers, statin). ACEI: angiotensin converting enzyme inhibitor; ARB: angiotensin receptor blocker; MACEs: major adverse cardiovascular events; MI: myocardial infarction; OR: odd ratio. (TIF) [file pone.0163068.s003.tif]

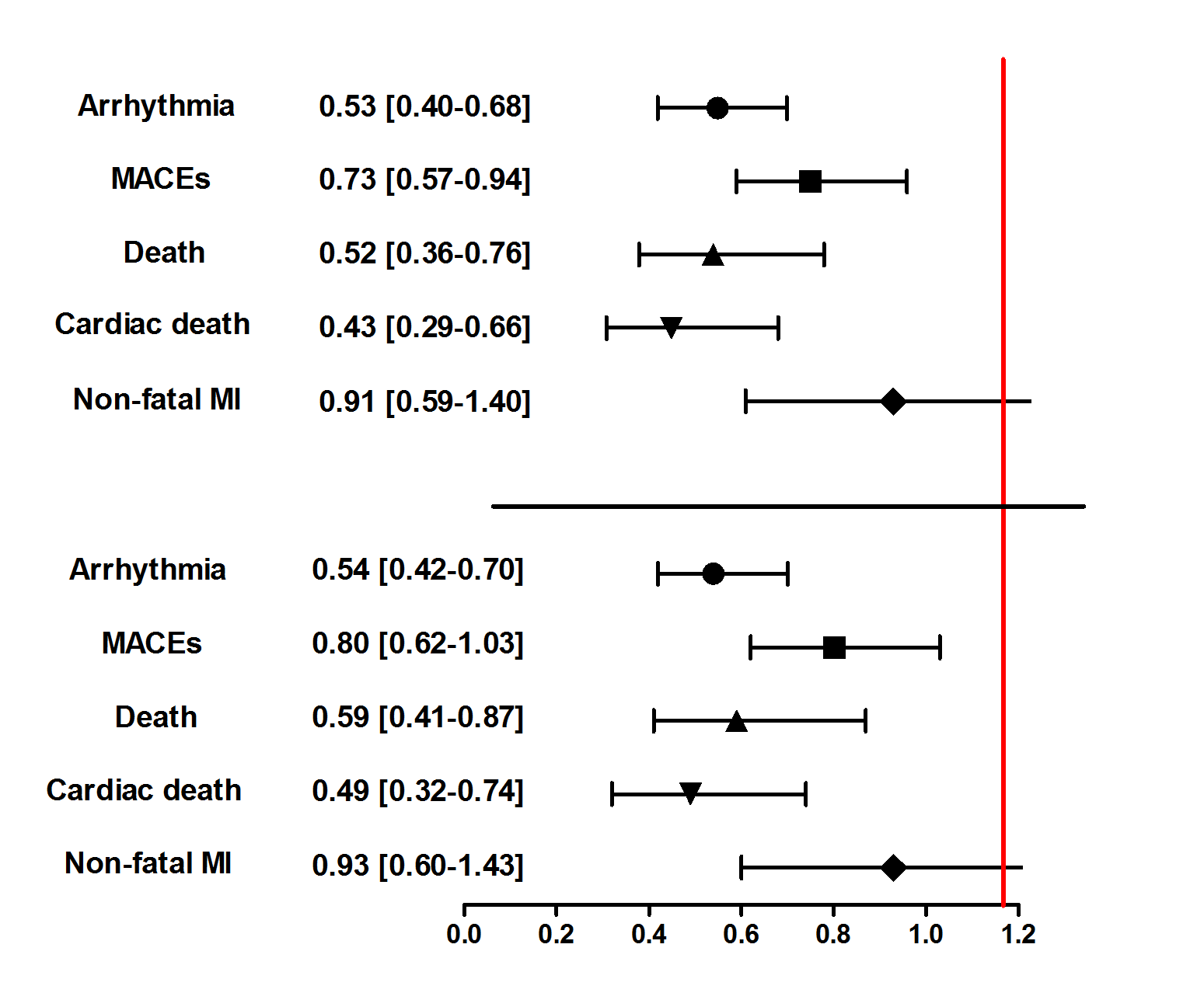

Supplement: S4 Fig — The top part shows ORs in Table 2; the bottom part shows ORs with additional adjusted for in-hospital treatment including reperfusion therapy (thrombolytic therapy, percutaneous coronary intervention, coronary artery bypass grafting) and medications (antiplatelet agents, ACEI/ARB, beta-blockers, statin). ACEI: angiotensin converting enzyme inhibitor; ARB: angiotensin receptor blocker; MACEs: major adverse cardiovascular events; MI: myocardial infarction; OR: odd ratio. (TIF) [file pone.0163068.s004.tif]

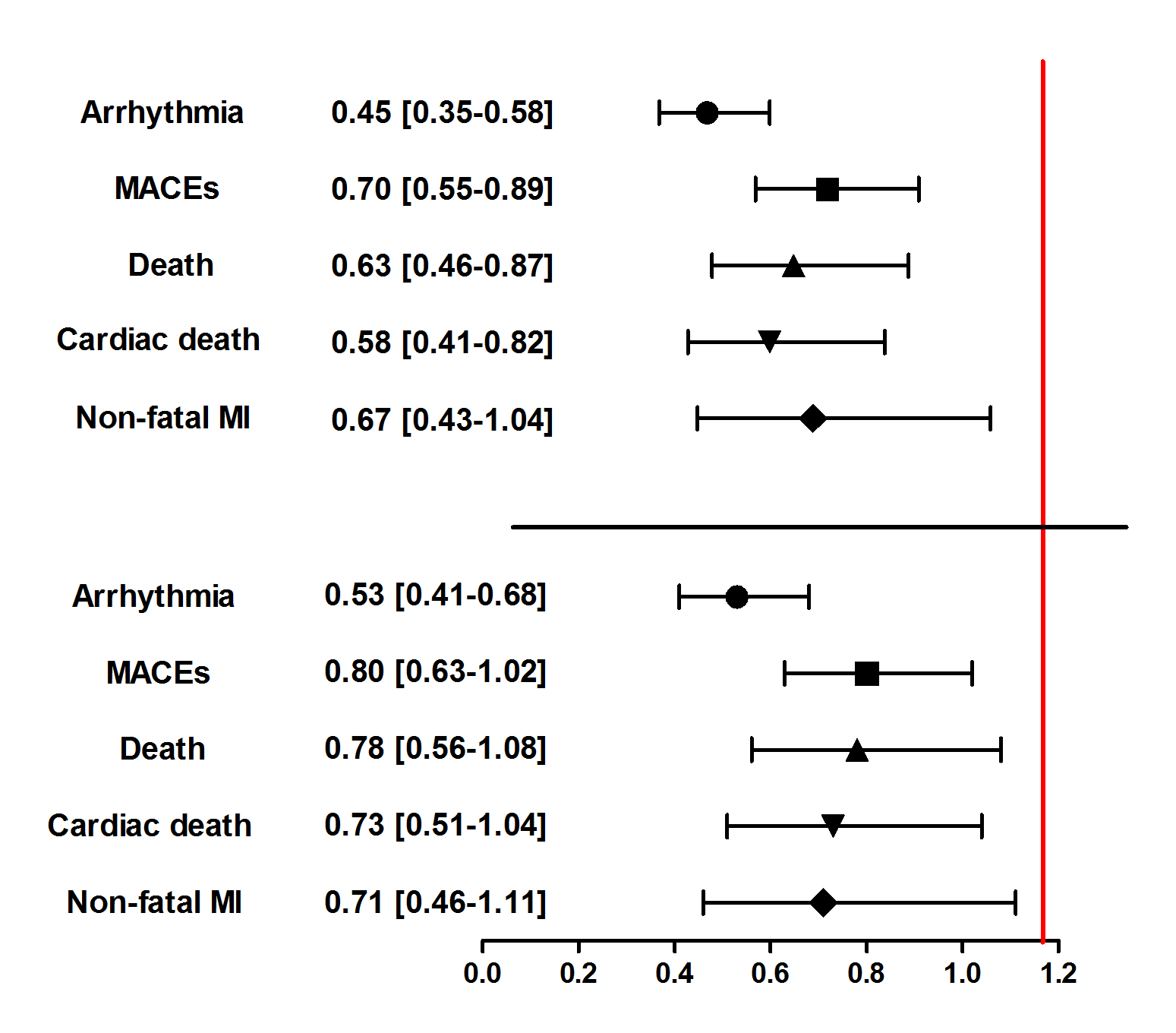

Supplement: S5 Fig — The top part shows ORs in Table 2; the bottom part shows ORs with additional adjusted for in-hospital treatment including reperfusion therapy (thrombolytic therapy, percutaneous coronary intervention, coronary artery bypass grafting) and medications (antiplatelet agents, ACEI/ARB, beta-blockers, statin). ACEI: angiotensin converting enzyme inhibitor; ARB: angiotensin receptor blocker; MACEs: major adverse cardiovascular events; MI: myocardial infarction; OR: odd ratio. (TIF) [file pone.0163068.s005.tif]
