# Supplementary material for: Impact of Prior Use of Four Preventive Medications on Outcomes in Patients Hospitalized for Acute Coronary Syndrome--Results from CPACS-2 Study
Source: PLoS One. 2016 Sep 14;11(9):e0163068. doi: 10.1371/journal.pone.0163068 (PMC5023149; doi:10.1371/journal.pone.0163068)
Supplement: S1 Table — (DOCX) [file pone.0163068.s006.docx]

Table S1. Detail definitions of outcomes

| Outcomes | Definition |
| --- | --- |
| Hypotension | Systolic blood pressure <90 mmHg measured at first present to emergency room or hospital |
| Tachycardia | Heart rate>=100 beats/min measured at first present to emergency room or hospital |
| Major arrhythmia | Persistent ventricular tachycardia or ventricular fibrillation or Mobitz type II or III atrio-ventricular block happened in hospital |
| MACEs | Include all-cause mortality, non-fatal new or reoccurred MI, and non-fatal stroke |
| All-cause mortality | Died in hospital or within 48 hours after discharge |
| New or reoccurred MI | Acute MI was defined in accordance with the European Society of Cardiology/American College of Cardiology 2000 consensus definition. For patient admitted as acute myocardial infarction, if the following criteria are satisfied, the patient is considered as having in hospital re-infarction:   \| Clinical scenario \| Criteria \| \| --- \| --- \| \| Recurrent symptoms occurring within 18 hours \| Chest pain lasting >=30 minutes and >=2 mm of ST elevation \| \| Recurrent symptoms occurring after lasting 18 hours \| CK rise to >2×ULRR and>50% above previous baseline value  or CK-MB value>ULRR and>50% above previous baseline value or new left bundle branch block or new Q waves \| \| Following percutaneous coronary intervention \| CK rise to>3×ULRR or CK-MB value>3×ULRR or new left bundle branch block or new Q waves \| \| Following coronary artery bypass grafting \| CK rise to>5×ULRR or CK-MB value>5×ULRR or new left bundle branch block or new Q waves \| |
| Stroke | There are signs or symptoms concordant with stroke happened in hospital, and the diagnosis was verified by computed tomography or magnetic resonance imaging |
| Major bleeding | Intracranial haemorrhage or a >5 g/dL decrease in haemoglobin concentration or a >15% absolute decrease in haematocrit |

CK: creative kinase; CK-MB: creative kinase MB; MACEs: major adverse cardiovascular events; MI: myocardial infarction; ULRR: upper limit of the reference range;
