# Supplementary material for: Impact of Prior Use of Four Preventive Medications on Outcomes in Patients Hospitalized for Acute Coronary Syndrome--Results from CPACS-2 Study
Source: PLoS One. 2016 Sep 14;11(9):e0163068. doi: 10.1371/journal.pone.0163068 (PMC5023149; doi:10.1371/journal.pone.0163068)
Supplement: S2 Table — (DOCX) [file pone.0163068.s007.docx]

Table S2. Baseline characteristics of included and excluded ACS patients

| Variables | Included  (N=14790) | Excluded  (N=351) | P |
| --- | --- | --- | --- |
| Age, Mean (sd) | 63.9 (11.8) | 64.6 (12.1) | 0.26 |
| Male | 69.5 | 65.7 | 0.13 |
| Health insurance | 82.6 | 89.2 | 0.00 |
| Risk factors of CVD |  |  |  |
| Current smoker | 31.1 | 12.3 | 0.00 |
| Hypertension | 57.4 | 35.6 | 0.00 |
| Diabetes | 20.5 | 11.1 | 0.00 |
| History of CVD |  |  |  |
| Myocardial infarction | 12.2 | 6.6 | 0.00 |
| Angina pectoris | 41.3 | 23.9 | 0.00 |
| Stroke/ transient ischemic attack | 10.1 | 6.6 | 0.00 |
| Heart failure | 6.2 | 2.9 | 0.00 |
| Reperfusion therapy |  |  |  |
| Percutaneous coronary intervention | 46.6 | 29.9 | 0.00 |
| Coronary artery bypass grafting | 0.9 | 0.9 | 1.00 |
| Thrombolytic | 6.3 | 5.4 | 0.51 |
| In-hospital medications |  |  |  |
| Antiplatelet | 99.0 | 97.4 | 0.00 |
| ACEI/ARB | 79.1 | 65.8 | 0.00 |
| Statin | 93.8 | 94.0 | 0.87 |
| Beta-blockers | 78.3 | 74.6 | 0.10 |

All data in the table are shown as %, except for age.

ACEI: angiotensin converting enzyme inhibitor; ACS: acute coronary syndrome; ARB: angiotensin receptor blocker; CVD: cardiovascular disease
